# Evaluation of Bacteriophage vB_SalS_KY05 Lysate on Physiological Status, Egg Production, and Intestinal Microbiota in Laying Hens

**DOI:** 10.3390/ani16213351

**Published:** 2026-10-23

**Authors:** Hsiao-Hui Huang, Wun-Yuan Wu, Yuan-Yu Lin

**Affiliations:** 1 Department of Animal Science and Technology, National Taiwan University, Taipei City 106, Taiwan; r13626004@ntu.edu.tw; 2 Department of Biology, University of Copenhagen, 2200 Copenhagen, Denmark; pvb630@bio.ku.dk

**Keywords:** safety evaluation, laying hens, intestinal health, bacteriophage lysate

## Abstract

Bacteriophage vB_SalS_KY05 (KY05) was propagated using a non-pathogenic *E. coli* K-12 strain. The lysate was administered via water for 56 days. Thirty Lohmann Brown-Classic laying hens (53 weeks old) were randomly assigned to three groups: the Control group (without phage lysate), the Low-Phage group (10^5^ PFU/mL) and the High-Phage group (10^8^ PFU/mL). This study aimed to evaluate the safety profile of the administered KY05 lysate by assessing its endotoxin content and its effects on immune responses and gut microbiota in laying hens. Phage lysate did not adversely affect physiological status, laying performance, plasma biochemical parameters, intestinal morphology, or inflammatory cytokine concentrations when compared with the results for the Control group, supporting the physiological safety of KY05 lysate in laying hens. Fecal *Lactobacillus* counts were not affected, while fecal *E. coli* counts showed a slight, although not statistically significant, decrease in the phage-treated groups. In cecal 16S rRNA analysis, microbial composition did not differ significantly among treatments. These findings support the safety of prolonged KY05 lysate administration under the experimental conditions used in this study and warrant further evaluation under field conditions.

## Simple Summary

The integrity of the intestine plays a crucial role in animal development and physiological function. A bacteriophage (phage) is a virus that specifically infects bacteria, and it may adhere to mucosal surfaces and eliminate the bacterial pathogens in the gastrointestinal tract. The phage lysates are typically considered highly efficient and cost-effective for large-scale production. However, phage lysates may contain residual endotoxin, triggering immune responses in animals, with potential impacts on intestinal microbiota. This study evaluated the administration of phage lysate in laying hens, resulting in no adverse impacts on physiological status, laying performance or intestinal microbiota. This suggested that phage lysate propagated using a non-pathogenic host may be a safe phage-derived additive for use in the poultry industry.

## 1. Introduction

Poultry plays a critical role in global food security, providing affordable animal protein through efficient production of meat and eggs [1]. *Salmonella* is one of the major foodborne pathogens associated with egg contamination [2]. However, the use of antibiotics during the egg production period is highly restricted due to the concern regarding the presence of antibiotic residues in eggs. Therefore, effective strategies for controlling *Salmonella* contamination in laying hens are essential for egg safety and public health. Bacteriophages (phages) are viruses that specifically infect bacteria and thus, are considered a potential alternative to antibiotics. The advantages of phages include their high host-specificity, allowing them to recognize specific receptors on the bacteria [3,4], and their self-replication at the site of infection (auto-dosing). Phages offer practical advantages such as ease of isolation, large-scale production, and adaptability [4,5,6,7]. These characteristics may comprise a cost-effect and flexible strategy for bacterial biocontrol [8].

Bacteriophage lysate is a primary product of phage lysis, containing free-phage particles and lysed bacterial components. The production of phage lysates is relatively rapid and cost-effective [9]; however, phage lysates often contain substantial impurities, particularly endotoxin. The presence of lipopolysaccharide (LPS), an endotoxin from the outer membrane of Gram-negative bacteria, is considered one of the major limitations of phage application due to its strong pro-inflammatory effect [10].

Bacteriophage vB_SalS_KY05 (KY05) is a polyvalent bacteriophage previously isolated from a commercial poultry farm in Taiwan [11]. KY05 exhibits a broad host range, making it capable of infecting multiple bacterial strains, such as *Salmonella* Typhimurium, and *E. coli* K12. Previous studies demonstrated that KY05 effectively reduced *Salmonella* colonization in chicks [11]. Yet, the impact of long-term administration of KY05 lysate on host immune status and intestinal microbiota composition of laying hens remains unknown. Therefore, our study orally administered KY05 lysate via drinking water in laying hens, aiming to evaluate the safety profile of KY05 lysate by determining its endotoxin content and to investigate the effects of KY05 lysate on the immunological parameters and microbiota composition of laying hen.

## 2. Materials and Methods

### 2.1. Bacteriophage Lysate Preparation and Examination

KY05 was propagated with liquid media in a 500 mL flask. Briefly, mid-log phase culture (O.D. 600 ≈ 0.6) of *E. coli* K12 was prepared in tryptic soy broth (TSB; HiMedia, Mumbai, India). The propagation was incubated at 37 ◦C overnight. After incubation, the phage lysate was collected by centrifugation at 5000 rpm for 10 min; the supernatant was filtered with 0.22 µm pore-size filters (Millipore Sigma, Burlington, MA, USA) and stored at 4 °C. The phage titer was determined by 10-fold serial dilution, followed by plaque assays and double-layer assays (DLA). The endotoxin level of KY05 lysate was determined using a ToxinSensor™ Chromogenic Limulus Amebocyte Lysate (LAL) Endotoxin Assay Kit (GenScript, Piscataway, NJ, USA), following the manufacturer’s instructions.

### 2.2. Animal Housing and Experimental Design

The animal experiment was approved by the Institutional Animal Care and Use Committee (IACUC) of National Taiwan University (approval number: NTU-115-EL-00042). During the 56-day experiment, hens were housed in individual cages (33 × 48 × 52 cm) and had ad libitum access to feed and water. Commercial feed (No. 12696, Formosa Fortune Group, Yilan, Taiwan) was provided during study. Hens were maintained under a 16L:8D photoperiod.

A total of 30 Lohmann Brown-Classic laying hens (53 weeks old) were weighed and randomly assigned to the following experimental groups: (1) Control, reverse osmosis (RO) water without KY05 lysate; (2) Low-Phage, RO water with 10^5^ PFU/mL KY05 lysate; and (3) High-Phage, RO water with 10^8^ PFU/mL KY05 lysate. Phage lysate supplements were prepared daily, and water residual volume was recorded by weighing.

### 2.3. Laying Performance and Egg Quality

Egg production rate and egg weight were record daily. The laying performance was evaluated based on laying rate, egg mass and feed efficiency. Laying rate was calculated as a percentage of eggs produced/hen/day. Egg mass (g/hen/day) was calculated as the average egg weight (g) × laying rate (%)/100. Feed efficiency (egg/feed) was calculated. Two eggs per hen were collected biweekly for egg quality analysis. The eggshell strength, albumen height, Haugh unit and yolk color were evaluated using a digital egg tester (DET6000, Nabel Co., Ltd., Kyoto, Japan).

### 2.4. Biological Sample Collection

At the end of study, the chickens were euthanized. Liver, spleen, small intestine, and cecal contents were collected. The weight of the liver and spleen were recorded. The relative organ weight (%) was calculated as the ratio of organ weight and body weight of the hens. Blood samples were collected from the wing vein with 22-gauge, 1-inch-long needle. The whole blood was collected into a heparin anticoagulant tube (BD Vacutainer^®^, Franklin Lakes, NJ, USA). Blood samples were centrifuged at 2000 rpm for 10 min to obtained the plasma. The plasma sample was used for cytokine and biochemical parameter analysis. Feces were examined for microbial counts. The cecal content of each hen was collected immediately after euthanasia for 16s rRNA analysis.

### 2.5. Biochemical Parameters and Pro-Inflammatory Cytokine Analysis of Chicken Plasma

Heparinized plasma samples were examined by biotechnology services (Bio-Cando Biotechnology Inc., Taipei, Taiwan) with a Clinical Analyzer 7180 (Hitachi High-Technologies Corp., Tokyo, Japan). The parameters measured were alanine aminotransferase (ALT), aspartate aminotransferase (AST), total protein (TP), and albumin (ALB). Globulin was defined by subtracting ALB from TP (globulin = TP–ALB). Immunoglobulin Y (IgY) was measured using a commercial competitive enzyme-linked immunosorbent assay (ELISA) kit (E12I0058, Bluegene, Shanghai, China). Plasma cytokine concentrations were determined using ELISA kits for interleukin (IL)-1β (E12I0010), IL-6 (E12I0006), and tumor necrosis factor (TNF)-α (E12T0008) (Bluegene, Shanghai, China). All assays were performed according to the manufacturers’ instructions.

### 2.6. Intestinal Morphology

The jejunum and ileum were collected immediately after euthanasia. The intestinal segments were rinsed with clean phosphate buffered saline (PBS) to remove the blood and residual contents, followed by fixation with 10% formaldehyde (Sigma-Aldrich, St. Louis, MO, USA). The tissues sections were stained with hematoxylin and eosin stain (H&E). The histological observations were conducted via an Olympus BX53 optical microscope (Olympus Corporation, Shinjuku, Tokyo, Japan). Villus height (VH) and crypt depth (CD) were measured using Olympus cellSens Standards software Version 1.16 (Olympus Corporation, Shinjuku, Tokyo, Japan). The measurements of each sample were obtained from three randomized fields. The villus height to crypt depth ratio (VH:CD) was calculated.

### 2.7. Fecal Microbial Count and Cecal 16S rRNA Sequencing Analysis

*Lactobacillus*, *E. coli*, and *Salmonella* counts were examined using detection pads (MC Media Pad, JNC, Kagoshima, Japan). Briefly, 1 mL of diluted supernatant of the feces sample was applied to the detection pad; for *E. coli* and *Salmonella*, the pads were incubated at 37 °C for 24 h; for *Lactobacillus*, the pads were incubated at 37 °C for 48 h. The microbial abundance was expressed in colony-forming units (CFU)/g.

Cecal DNA was extracted with the QIAamp^®^ Fast DNA Stool Mini Kit (Qiagen, Hilden, Germany). The total cecal DNA samples were analyzed via full-length 16S rRNA sequencing analysis using PacBio platforms (PacBio, Menlo Park, CA, USA). Sequence denoising and amplicon sequence variant (ASV) inference were conducted using the Divisive Amplicon Denoising Algorithm 2 (DADA2), which included quality filtering, error correction and chimera removal. Taxonomic classification was performed using QIIME2 with the SILVA (version 138.2) database. Species richness was estimated using observed features and Chao1 indices, while evenness was assessed using the Shannon and Gini–Simpson indices. Principal coordinate analysis (PCoA) of beta diversity between samples was calculated by Bray–Curtis Dissimilarity and weighted UniFrac metrics; the significant difference of beta diversity was assessed using permutational multivariate analysis of variance (PERMANOVA). The identical taxa among groups were identified using Linear Discriminant Analysis Effect Size (LEfSe) Taxa, with *p* < 0.05 and an LDA score > 2.0 considered significant biomarkers.

### 2.8. Statistical Analysis

Data are presented as mean ± standard deviation (SD). Normality and homogeneity of variance were assessed using the Shapiro–Wilk test. For data that met the assumptions of normality and homogeneity of variance, one-way analysis of variance (ANOVA), followed by Tukey’s post hoc test, were performed to compare groups. When the assumptions were not met, the Kruskal–Wallis test, followed by Dunn’s multiple-comparison test, were used, as appropriate. Statistical analyses and graph preparation were performed using GraphPad Prism version 10 (GraphPad Software, San Diego, CA, USA). Differences were considered statistically significant at *p* < 0.05.

## 3. Results

### 3.1. Profile of Phage KY05 Lysate and Water Intake of Laying Hens

The average phage KY05 titer in KY05 lysate was 1.40 ± 0.36 × 10^10^ PFU/mL. Endotoxin levels in the phage lysate were assessed using an LAL assay, and randomly sampled preparations contained an average endotoxin concentration of 8.68 ± 0.97 × 10^4^ EU/mL. The effect of the administration of phage KY05 on water intake was recorded. The average intake was approximately 150 to 220 mL/hen/day. The average daily water intake was 190.5 ± 17.07 mL, 188.6 ± 19.46 mL, and 195.1 ± 16.30 mL/hen/day for the Control, Low-Phage, and High-Phage groups. No differences were detected among the groups (*p* > 0.05).

### 3.2. Effects of KY05 Lysate on Laying Performance and Egg Quality 

The effects of KY05 lysate on laying performance and egg quality are shown in Table 1. During the 8-week experiment, feed intake and egg weight were similar among groups. The High-Phage group exhibited a higher egg production rate than that of the Control and Low-Phage groups. Similar result was observed for egg mass, with both the Control and High-Phage groups showing significantly higher results than those for the Low-Phage group (*p* < 0.0001). For feed efficiency (egg/feed), no significant difference was detected between the Control and Low-Phage groups. However, both groups exhibited significantly higher feed efficiency than that of the High-Phage group (*p* < 0.0001). Moreover, no significant differences in any of the egg quality parameters were observed among treatments (*p* > 0.05).

### 3.3. Immune-Organ Weight

The effects of KY05 lysate on body and immune-organ weight are shown in Table 2. The average initial body weights of laying hens for the Control, Low-Phage and High phage groups were 1.93 kg, 1.96 kg and 1.95 kg. No significant differences in body, liver, and spleen weight were observed among groups (*p* > 0.05). A significant difference was shown in regards to relative spleen weight by the Kruskal–Wallis test (*p* = 0.048); however, none of the pairwise comparisons following Dunn’s multiple-comparison test reached statistical significance (adjusted *p* > 0.05).

### 3.4. Plasma Biochemical Parameters

The effects of KY05 lysate on body weight and immune-organ weight are shown in Table 3. The analysis was conducted for liver function or immune-related biochemical parameters. Briefly, there were no statistically significant differences among AST, ALT, TP, ALB and IgY observed in either the Low-Phage or High-Phage group compared with the results for the Control group (*p* > 0.05).

### 3.5. Pro-Inflammatory Cytokine Levels in Plasma and Ileal Tissue

**Figure 1:**
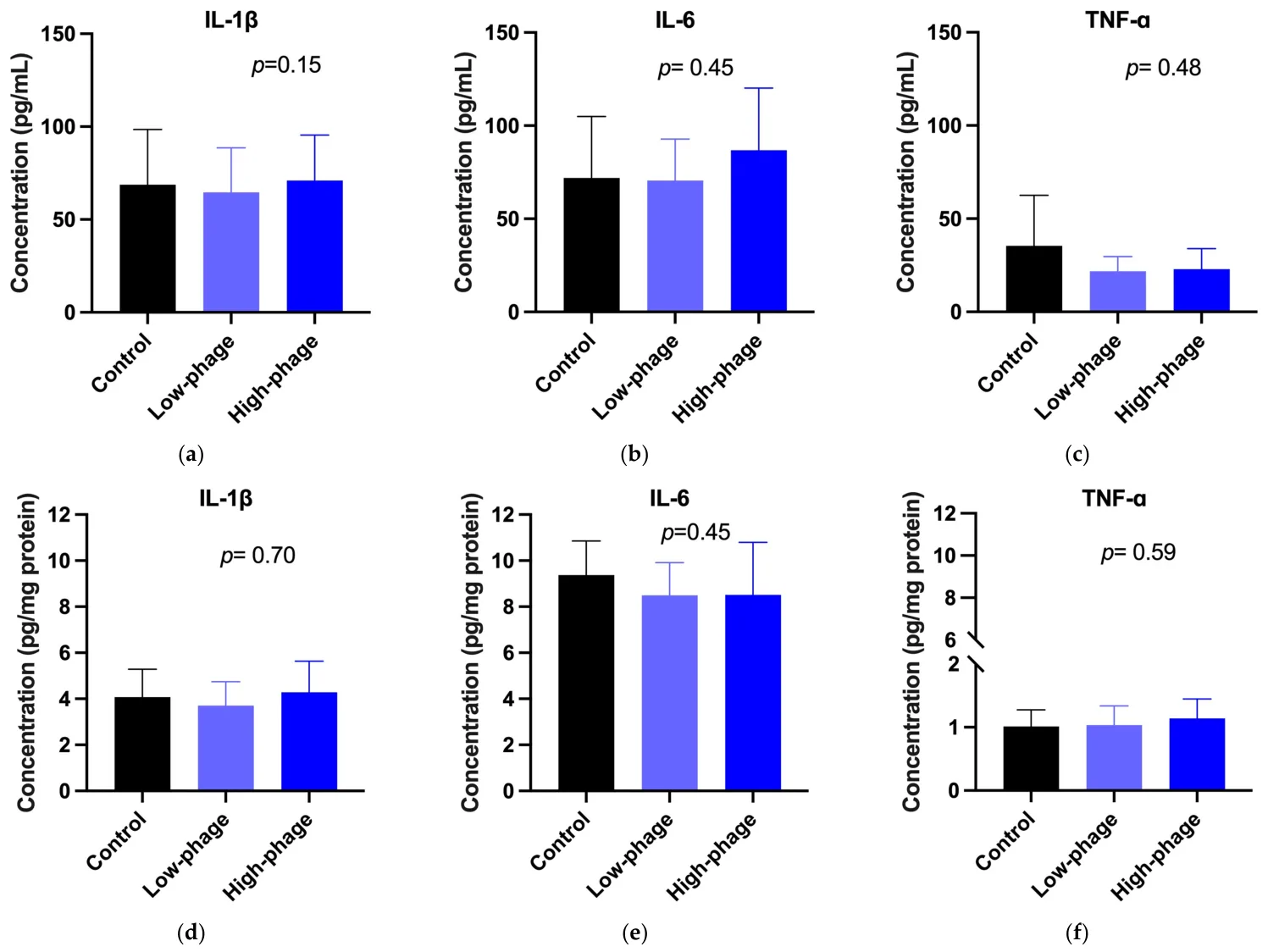
Effects of KY05 lysate on pro-inflammatory cytokine levels. (**a**) IL-1β level in plasma; (**b**) IL-6 level in plasma; (**c**) TNF-α level in plasma; (**d**) IL-1β level in ileum tissue; (**e**) IL-6 level in ileum tissue; and (**f**) TNF-α level in ileum tissue. Results expressed as mean ± SD (*n* = 10 per group). Statistical analysis was performed using one-way ANOVA, followed by Tukey’s post hoc test. Differences were considered statistically significant at *p* < 0.05. Control: RO water without KY05 lysate; Low-Phage: RO water with 10^5^ PFU/mL KY05 lysate; and High-Phage: RO water with 10^8^ PFU/mL KY05 lysate. Colors represent the three experimental groups.

The effects of KY05 lysate on cytokine concentrations in plasma and ileal tissue are presented in Figure 1. In both plasma and ileal tissue, no significant differences in the concentrations of the pro-inflammatory cytokines IL-1β, IL-6, and TNF-α (*p* > 0.05) were observed among the Control, Low-Phage, and High-Phage groups. The cytokine concentrations and associated variations remained at similar levels across all treatments.

### 3.6. Integrity of Jejunum and Ileum

**Figure 2:**
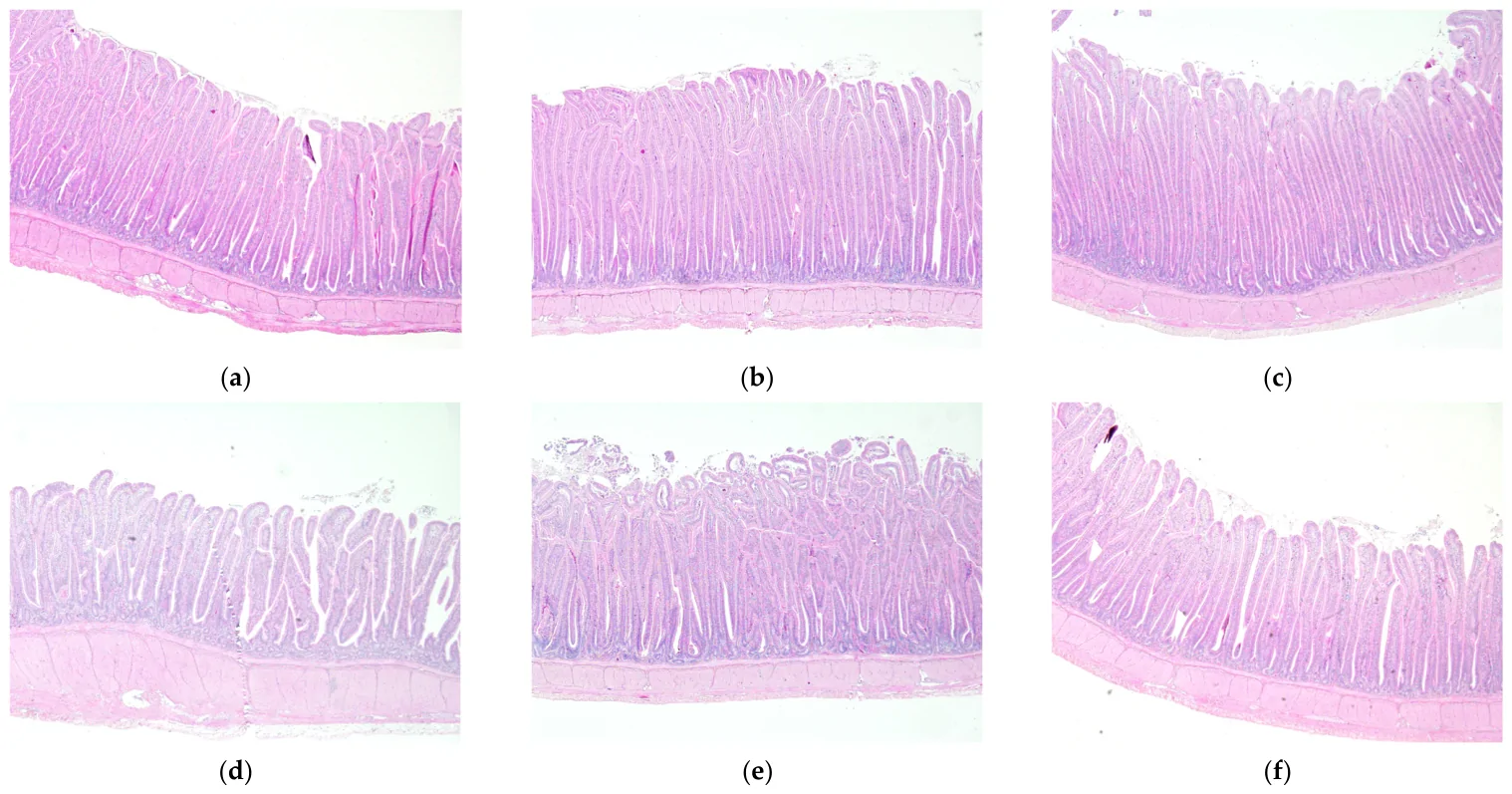
Histological section of jejunum of (**a**) Control; (**b**) Low-Phage; (**c**) High-Phage groups. Histological section of ileum of (**d**) Control; (**e**) Low-Phage; (**f**) High-Phage groups. Control: RO water without KY05 lysate; Low-Phage: RO water with 10^5^ PFU/mL KY05 lysate; and High-Phage: RO water with 10^8^ PFU/mL KY05 lysate.

The representative histological sections of the jejunum and ileum are shown in Figure 2. The histological evaluations were measured as VH, CD, and VH:CD. The effects of KY05 lysate on villus integrity are summarized in Table 4. No significant differences were observed in the jejunum and ileum among groups (*p* > 0.05).

### 3.7. Fecal Lactobacillus and E. coli Count

**Figure 3:**
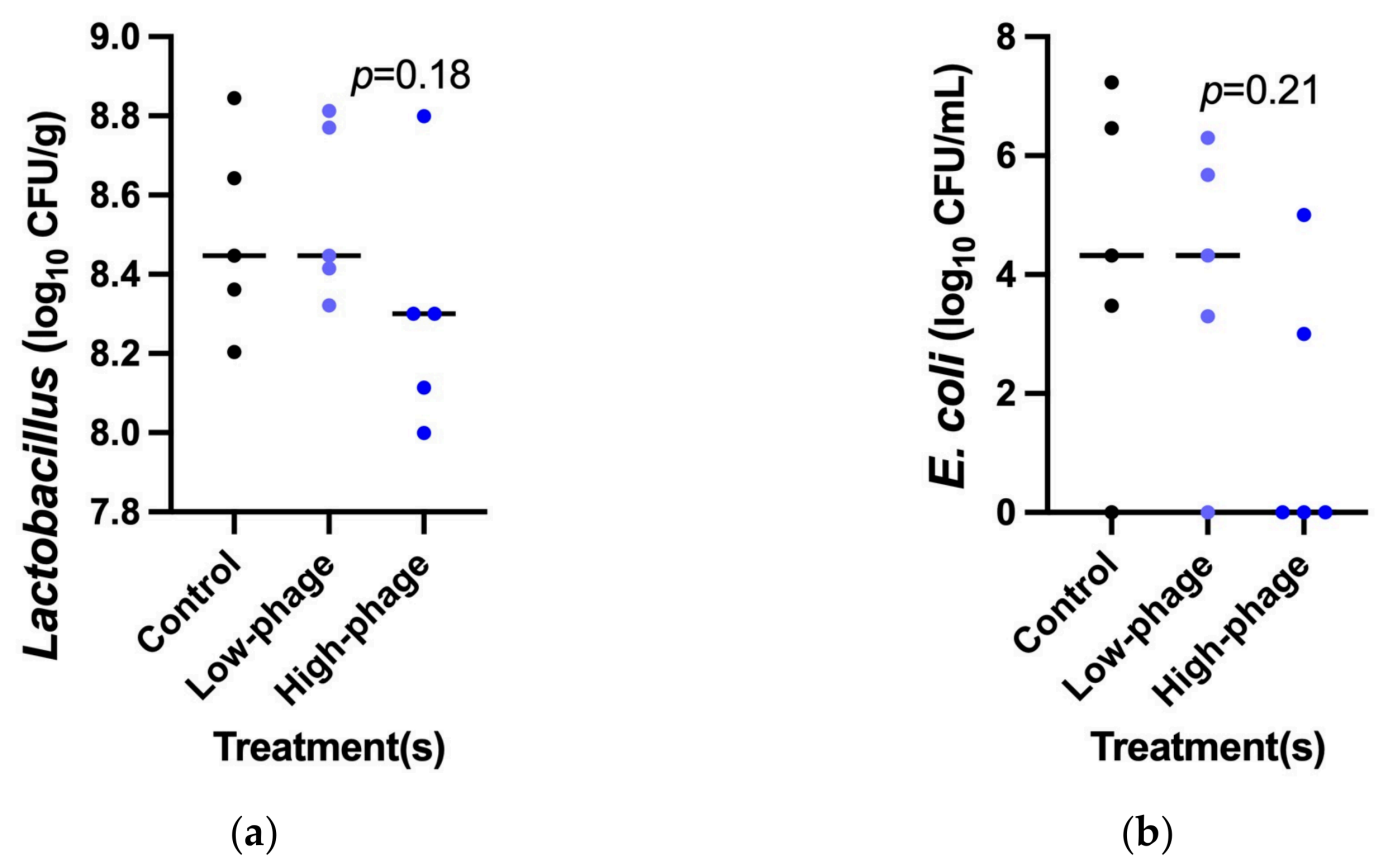
Effects of KY05 lysate on fecal microbial counts of laying hens. (**a**) Fecal *Lactobacillus* counts and (**b**) *E. coli* counts. Scatter plots show individual data points, with horizontal lines representing the median (*n* = 10 per group). Statistical analysis was performed using the Kruskal–Wallis test with Dunn’s post hoc test. Differences were considered statistically significant at *p* < 0.05. Control: RO water without KY05 lysate; Low-Phage: RO water with 10^5^ PFU/mL KY05 lysate; and High-Phage: RO water with 10^8^ PFU/mL KY05 lysate. Colors represent the three experimental groups.

The *Lactobacillus*, *Salmonella* and *E. coli* counts of the fecal samples were detected immediately after collection. The effects of KY05 lysate on the microbial counts is presented in Figure 3. No *Salmonella* colonies were detected in any excreta samples throughout the examination; therefore, no quantitative results are presented. There were no significant differences in *Lactobacillus* counts among groups. Likewise, the enumeration of *E. coli* showed no significant differences among treatment groups.

### 3.8. Cecal Microbiota Composition and Diversity

**Figure 4:**
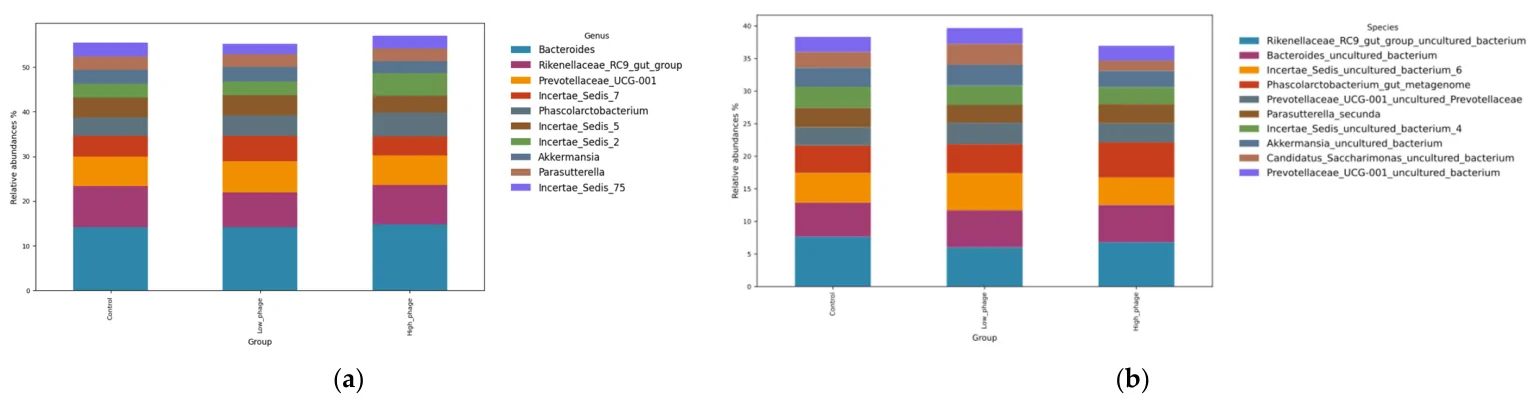

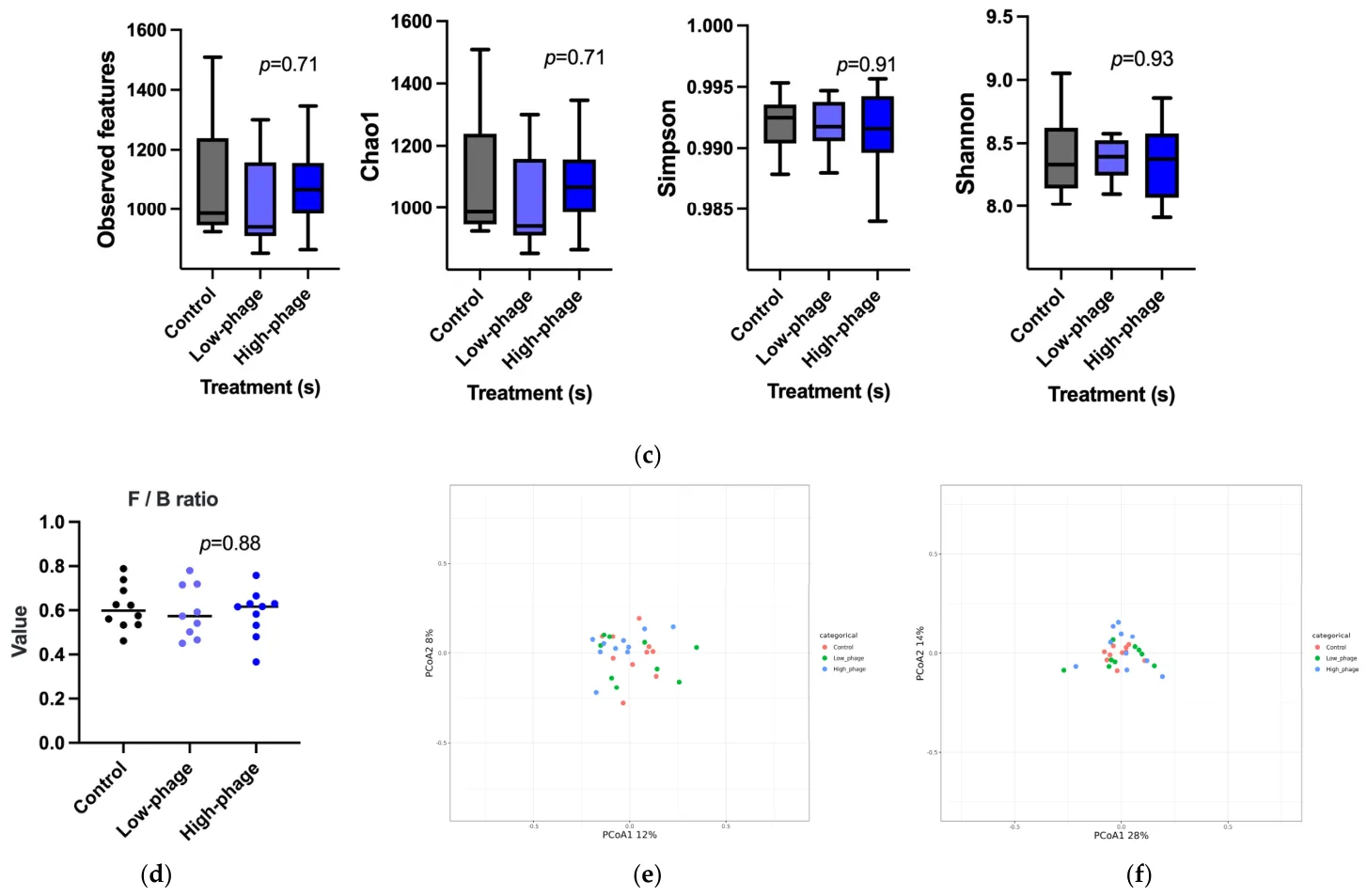
Effects of KY05 lysate on microbiota diversity and composition in laying hens. Sample sizes were *n* = 10, 9, and 10 for the Control, Low-Phage, and High-Phage groups, respectively. (**a**) Top 10 microbial compositions at the genus level; (**b**) top 10 microbial compositions at the species level; (**c**) alpha diversity indices: observed feature, Chao1, Simpson index and Shannon index. Box plots extend from the minimum to the maximum values, with horizontal lines indicating the median.; (**d**) *Firmicutes*-to-*Bacteroidetes* (F/B) ratio. Scatter plots show individual data points, with horizontal lines representing the median; (**e**) PCoA using Bray–Curtis Dissimilarity (PERMANOVA: *p* = 0.12, R2 = 0.05); (**f**) PCoA using weighted UniFrac distances (PERMANOVA: *p* = 0.21, R2 = 0.03). Control: RO water without KY05 lysate; Low-Phage: RO water with 10^5^ PFU/mL KY05 lysate; and High-Phage: RO water with 10^8^ PFU/mL KY05 lysate. Colors represent the three experimental groups.

The relative abundance and diversity of microbiota are shown in Figure 4. The dominant microbial taxa were Bacteroides, Rikenellaceae, and Prevotellaceae at the genus and species level. No significant differences were observed among groups for alpha diversity (*p* > 0.05), which indicates similar richness and evenness within samples. Beta diversity was evaluated using Bray–Curtis Dissimilarity, with phylogeny-based weighted UniFrac distances used to assess microbial community structure. Based on the results of PCoA and PERMANOVA, no significant differences in beta diversity were observed for either Bray–Curtis Dissimilarity or weighted UniFrac distances among groups (*p* > 0.05).

**Figure 5:**
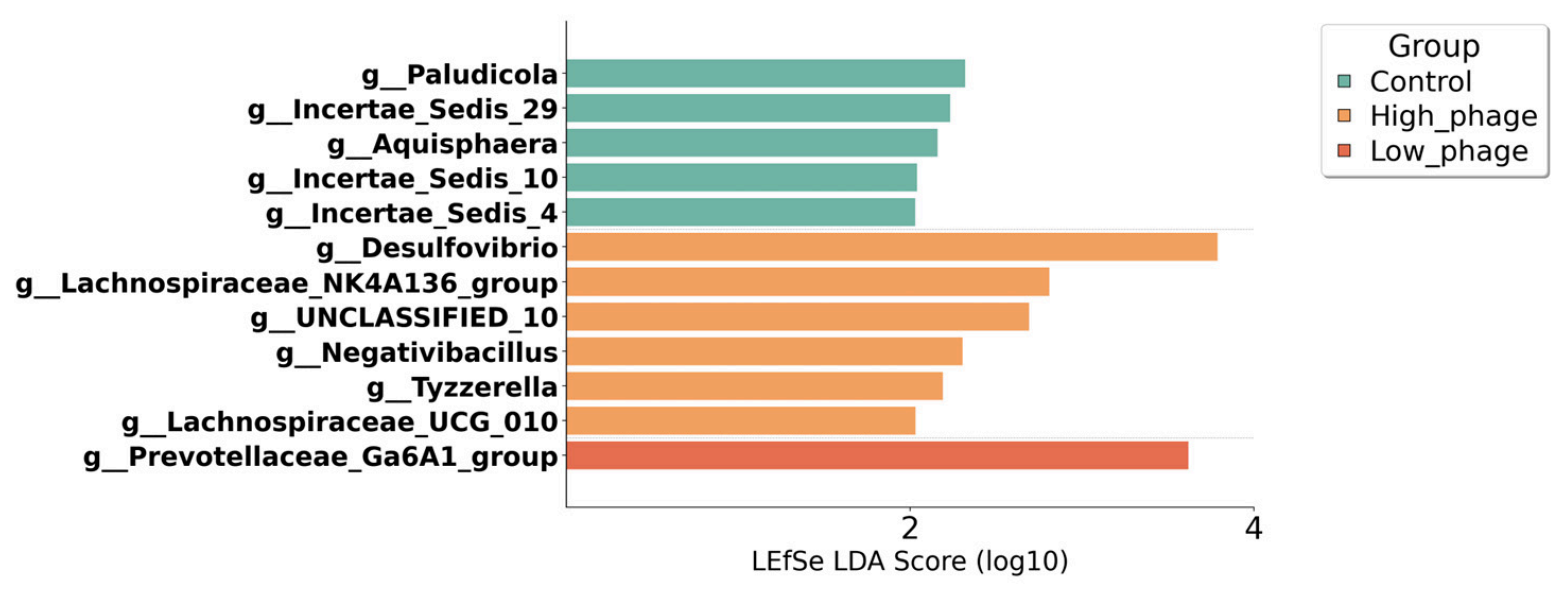
LEfSe analysis of identified biomarker in each group. Taxa with a Linear Discriminant Analysis (LDA) score > 2.0 were considered as identical biomarkers. Control: RO water without KY05 lysate; Low-Phage: RO water with 10^5^ PFU/mL KY05 lysate; and High-Phage: RO water with 10^8^ PFU/mL KY05 lysate.

LEfSe analysis identified bacterial biomarkers among groups. The identical taxa of each group are presented in Figure 5. The Control group was characterized by a higher abundance of *Lactobacillus johnsonii*. The Low-Phage group was enriched in *Prevotellaceae_Ga6A1_group*. In contrast, the High-Phage group showed enrichment in *Lachnospiraceae NK4A136 group*, along with several unclassified or Incertae sedis taxa, suggesting an increase in less-characterized microbial populations under high-dose phage treatment.

## 4. Discussion

### 4.1. Production and Endotoxin Characterization of KY05 Phage Lysate

Phage KY05 is a polyvalent bacteriophage originally isolated from a local poultry farm in Taiwan. KY05 exhibits a broad host range, capable of infecting both pathogenic bacteria (*Salmonella* Typhimurium) and non-pathogenic strains (*E. coli.* K-12) [11]. In our study, KY05 was propagated using log-phase non-pathogenic *E. coli.* K-12 as the host; the resulting titer of K12-propagated KY05 reached approximately 10^10^ PFU/mL. Comparable titers have also been reported in studies utilizing bioreactor-based phage propagation systems [12]. One of the major concerns of phage administration is the residual presence of bacterial endotoxins. Current Food and Drug Administration (FDA) guidelines recommend that endotoxin levels for non-intrathecal administration should not exceed 5 endotoxin units (EU)/kg of body weight/hour, whereas intrathecal administration should not exceed 2 EU/kg of body weight/hour [9,13,14,15]. The concentration of endotoxin in *E. coli.* K12-propagated KY05 lysate was approximately 8.68 ± 0.97 × 10^4^ EU/mL. According to Oliveira et al. [16], the lethal dose of *E. coli* endotoxins in chickens is 50 mg/kg, or 5 × 10^8^ EU/kg (with 1 EU/mL ≈10 ng/mL). Based on an estimated daily water intake of 200 mL and an average body weight of 2 kg per chicken, the calculated exposure was approximately 0.87 µg LPS/kg/day in the Low-Phage group and 0.87 mg LPS/kg/day in the High-Phage group. This level is comparable to doses reported in previous studies, where 1 mg LPS/kg body weight (LPS L2880 from *E. coli*) was used to induce immune responses [17].

### 4.2. Physiological Status and Production Performance Following KY05 Lysate Administration

During the 8-week experimental period, no consistent or systematic differences in water or feed intake were observed among treatment groups across different weeks. These results suggest that long-term administration of KY05 lysate via drinking water does not adversely affect drinking behavior or feed consumption in laying hens. Previous studies applying supplemented phage mixtures in feed also reported no adverse effects on laying performance and egg quality in laying hens [18,19]. Consistently, in the present study, no significant difference in laying rate was observed between the High-Phage and Control groups (*p* > 0.05), indicating that KY05 lysate does not negatively affect overall egg production performance and egg quality parameters under the current experimental conditions.

### 4.3. Immune Responses Following KY05 Lysate Administration

The phage-specific immune response may be induced in patients during phage treatment [20]. From an immunogenicity aspect, it has been reported that over 30 billion phages undergo transcytosis across the gut epithelium daily and can subsequently disseminate into the blood, lymphatic system, and various organs [21]. In chickens, the liver and spleen are two major organs closely associated with immune function; in particular, liver development is essential for maintaining normal immune function [22] and egg quality [23] in laying hens. Our results showed that the long-term administration of KY05 lysate did not significantly affect immune organ and plasma biochemistry, suggesting that liver function was not adversely impacted. Sarrami et al. [24] reported increased serum IgG and IgM levels in phage-fed groups, highlighting the strain-specific nature of host immune responses to bacteriophage administration. IgY is the predominant immunoglobulin in chickens [25], and our results showed decreasing IgY trends in phage-treated birds. This shift in immunoglobulin profiles may be influenced by IgM. Sarrigeorgiou et al. [26] reported increased anti-LPS IgM, which may be a key factor of animal mortality. Furthermore, phages appear to exert diverse effects on cytokine production, e.g., Majewska et al. [27] demonstrated that inflammatory responses were primarily observed at high phage concentrations (10^9^ PFU/mL). This phenomenon could be depended on the phage type [28]; thus, our study shows that KY05 lysate did not induce pro-inflammatory cytokine, highlighting the fact that the immunological effects of phages are highly strain- and context-dependent.

### 4.4. Intestinal Health and Fecal Microbiota Following KY05 Lysate Administration

The intestine plays a central role in nutrient utilization and immune-barrier function. Its development, structure, and physiological functions are highly dependent on diet composition [29,30]. In the present study, histological examination revealed normal intestinal architecture across all treatment groups. Villus height was typically highest in the duodenum, followed by the jejunum and ileum, with the lowest values observed in the ileum [31]. No obvious signs of epithelial damage, villus erosion, inflammatory cell infiltration, or tissue degeneration were detected in either phage-treated group compared with the results for the Control group. Similar findings were reported by Wanasawaeng et al. [32], who observed a significant increase in villus surface area in phage-treated groups compared with the controls (*p* < 0.05). In the present study, the VH:CD ratio showed no significant differences among groups (*p* > 0.05). It has been reported that reduced VH or increased CD may indicate decreased nutrient absorption capacity in the small intestine [33]. This work indicated that KY05 lysate supplementation does not adversely affect intestinal morphology or structural integrity under the conditions of this study.

Horizontal transmission of enteric pathogens in poultry is commonly associated with the ingestion of feces from infected carriers [34]. Other studies have reported increased *Lactobacillus* and decreased coliform bacteria in excreta under phage supplementation in non-challenged conditions [24]; however, in the present study, the abundance of *Lactobacillus* and *E. coli* in the fecal samples was not altered by KY05 lysate. Notably, no *Salmonella* was detected in any of the experimental groups, likely reflecting the relatively hygienic rearing conditions and the absence of an experimental *Salmonella* challenge. This is consistent with the work of Upadhaya et al. [35], who reported that under non-challenged and hygienic conditions, phage supplementation did not significantly affect *E. coli* or *Salmonella* populations. In contrast, *E. coli* counts showed a decreasing trend in phage-treated groups, although the differences were not statistically significant. Phage administration may exert subtle microbiota-modulating effects, even in the absence of pathogen challenge. However, microbiota responses to phage supplementation may be highly state-dependent, and host microbiota might influence phage efficacy by phage–bacteria interactions in vivo [36].

### 4.5. Cecal Microbiota Following KY05 Lysate Administration

A healthy gut microbiota generally plays an important role in modulating intestinal immune function and protecting against pathogen invasion [37]. Our study identified approximately 1000 to 1200 bacterial species across samples using full-length 16S rRNA sequencing. *Firmicutes* and *Bacteroidota* are typically dominant in the cecal microbiota of laying hens. An increased *Firmicutes*-to-*Bacteroidota* (F/B) ratio has been associated with obesity in animals [33]. Previous studies have reported that high egg-producing chickens exhibit higher abundances of *Firmicutes* [38]. However, in the present study, KY05 lysate administration did not significantly affect the F/B ratio among groups (*p* > 0.05).

Phage supplementation may selectively reduce pathogenic bacterial populations and thereby contribute to gut microbiota modulation [35]. Alpha diversity analysis showed consistently high diversity across all treatment groups, indicating high richness, evenness, and overall microbial stability. Similar findings have been reported by Wanasawaeng, Thomrongsuwannakij and Chansiripornchai [32], who observed no significant effects of phage treatment on alpha diversity following *Salmonella* challenge. This may indicate that KY05, due to its host specificity, does not substantially alter overall microbial richness or evenness. For beta diversity analysis, weighted UniFrac is a quantitative measurement that accounts for relative abundance changes in taxa [39]; the Bray–Curtis Dissimilarity and weighted UniFrac distance showed no significant differences based on PERMANOVA analysis (*p* > 0.05). These results suggest that KY05 administration did not significantly affect dominant microbial communities. Chen et al. [40] compared gut microbiota between high- and low-producing hens and found that differences in productivity were not driven by overall microbial richness or diversity but by the presence of specific bacterial taxa. Therefore, future analysis using LEfSe is warranted to identify potential biomarker taxa associated with each treatment group and to further elucidate phage-related microbial signatures. LEfSe is performed to identify potential biomarker taxa (LDA score > 2) associated with each treatment group. Bacteriophage administration may indirectly enhance SCFA-associated microbial functions and promote beneficial shifts in gut microbial activity [24]. *s__Prevotellaceae_Ga6A1_group* was identified in the Low-Phage group. *Prevotellaceae* are generally associated with carbohydrate metabolism and SCFA production, which are important for maintaining gut microbial balance [41]. In the High-Phage group, *g__Desulfovibrio* and *s__Lachnospiraceae_NK4A136_group* were identified as representative taxa. *Lachnospiraceae* are also well known for SCFA production and have been linked to improved intestinal barrier function and gut health [42]. This suggests that high-dose KY05 supplementation may be associated with shifts in low-abundance or previously uncharacterized microbial populations, KY05 may contribute to a more favorable gut environment.

### 4.6. Study Limitation and Future Perspectives

Although no significant adverse effects were observed following KY05 lysate administration, supporting its overall safety under the experiment of study, several limitations should be considered for further investigation. The present study was conducted under controlled experimental conditions using RO water, which may not fully reflect the physicochemical and microbiological characteristics of drinking water in commercial poultry farms. Future studies should therefore evaluate the stability, safety, and efficacy of KY05 lysate under field-relevant conditions, including tap-water systems and environments with greater microbial complexity. The 56-day experimental period may not be sufficient to determine the consequences of prolonged KY05 lysate administration. Longer-term studies are warranted to assess sustained effects on production performance, immune responses, intestinal barrier function, and gut microbiota. Furthermore, no formal a priori power analysis was performed to determine the sample size, which may have limited the study’s ability to detect small differences between groups. A sensitivity power analysis for the present design (three groups, n = 10 per group; α = 0.05; power = 0.80) indicated that only large effects (Cohen’s f ≥ 0.60; corresponding to approximately 8–15% changes in villus height, body weight and plasma biochemical parameters relative to the Control) could be reliably detected. Therefore, the lack of significant differences should not be interpreted as evidence of equivalence. Future studies should define a primary outcome and conduct an a priori power analysis to determine an adequate sample size.

Finally, combination strategies may provide an additional approach for improving pathogen control. Previous research has demonstrated synergistic effects between phages and natural antimicrobial compounds, such as thyme oil, against *Staphylococcus aureus* in chicken meat products [43]. Such findings support further investigations of whether KY05 lysate can be combined with other feed additives or antimicrobial agents to enhance pathogen control while maintaining host safety and intestinal health. These approaches may clarify the practical applicability of KY05 lysate as a potential antimicrobial strategy in commercial poultry production.

## 5. Conclusions

Our study presented the effects of bacteriophage KY05 lysate on laying hens, with the impact comprehensively evaluated across physiological performance, immune responses, and gut microbiota composition. KY05 lysate administration via drinking water did not adversely affect growth or laying performance, indicating that long-term administration is physiologically safe under the current experimental conditions. From an immunological perspective, KY05 lysate did not induce hepatic toxicity or systemic immune disruption, inflammatory pathology, or impairment of intestinal morphology. Microbiota analysis further demonstrated that KY05 lysate maintained overall microbial stability. In conclusion, KY05 lysate administration appears to be microbiologically and physiologically safe for laying hens under the tested conditions. While it does not significantly enhance production performance, it may exert modulatory effects on low-abundance microbial populations and functional metabolic potential. These findings provide a foundational basis for future studies to explore optimized dosing strategies, combination applications, and potential functional benefits of bacteriophage-based interventions in poultry production systems.

## Figures and Tables

**Table 1:** Effects of KY05 lysate on laying performance and egg quality of laying hens.

Items	Control	Low-Phage	High-Phage	*p* Value
Feed intake (g)	111.60 ± 15.29	108.92 ± 24.73	116.08 ± 11.96	0.128
Egg weight (g)	63.32 ± 1.30	63.28 ± 1.26	63.62 ± 1.85	0.446
Egg production rate (%)	92.73 ± 6.79 ^ab^	92.00 ± 8.82 ^b^	95.82 ± 5.99 ^a^	0.011
Egg mass (g/hen/day)	58.72 ± 4.47 ^ab^	57.97 ± 6.00 ^b^	60.60 ± 4.16 ^a^	0.014
Feed efficiency (egg/feed)	0.57 ± 0.02 ^a^	0.58 ± 0.03 ^a^	0.55 ± 0.03 ^b^	<0.0001
Shell strength (kgf)	5.23 ± 0.71	5.09 ± 1.39	4.86 ± 0.91	0.053
Albumin height (mm)	7.06 ± 0.97	7.51 ± 1.53	7.46 ± 0.96	0.193
Yolk color	5.61 ± 0.96	5.91 ± 1.62	5.89 ± 0.92	0.066
Haugh unit ^1^	82.63 ± 5.38	83.61 ± 10.68	84.60 ± 6.38	0.239

All measurements are presented as mean ± SD (*n* = 10 per group). Different lowercase letters indicate significant differences between treatments (*p* < 0.05). ^1^ Haugh unit = 100 × log (H − 1.7W^0.37^ + 7.6). Differences were considered statistically significant at *p* < 0.05.

**Table 2:** Effects of KY05 lysate on immune-organ weight of laying hens.

Items	Control	Low-Phage	High-Phage	*p* Value
Body weight (kg)	1.90 ± 0.20	1.94 ± 0.24	1.95 ± 0.18	0.815
Liver weight (g)	36.75 ± 3.81	33.70 ± 6.12	38.71 ± 4.11	0.080
Relative liver weight (%)	1.95 ± 0.25	1.75 ± 0.27	1.99 ± 0.26	0.091
Spleen weight (g)	1.56 ± 0.32	1.62 ± 0.31	1.38 ± 0.40	0.286
Relative spleen weight (%)	0.08 ± 0.01	0.08 ± 0.01	0.07 ± 0.02	0.048

Data expressed as mean ± SD (*n* = 10 per group). Statistical comparisons were performed using the Kruskal–Wallis test, followed by Dunn’s multiple-comparison test. Differences were considered statistically significant at *p* < 0.05. Control: RO water without KY05 lysate; Low-Phage: RO water with 10^5^ PFU/mL KY05 lysate; and High-Phage: RO water with 10^8^ PFU/mL KY05 lysate.

**Table 3:** Effects of KY05 lysate on plasma biochemical parameters and cytokine of laying hens.

Items	Control	Low-Phage	High-Phage	*p* Value
AST (U/L)	183.89 ± 12.6	187.18 ± 29.5	180.06 ± 17.0	0.751
ALT (U/L)	1.07 ± 0.46 **	1.06 ± 0.52 ***	1.16 ± 0.85 ***	0.936
TP (g/dL)	4.38 ± 0.53	3.96 ± 0.30	4.07 ± 0.42	0.092
ALB (g/dL)	2.21 ± 0.21	2.02 ± 0.18	2.05 ± 0.24	0.113
Globulin * (g/dL)	2.17 ± 0.37	1.94 ± 0.21	2.02 ± 0.24	0.204
IgY (mg/mL)	4.16 ± 1.64	2.45 ± 1.19	2.95 ± 1.79	0.047

Data expressed as mean ± SD (*n* = 10 per group). Differences were considered statistically significant at *p* < 0.05. AST, aspartate aminotransferase; ALT, alanine aminotransferase; TP, total protein; ALB, albumin; * Globulin = TP-ALB; IgY, immunoglobulin Y. Control: RO water without KY05 lysate; Low-Phage: RO water with 10^5^ PFU/mL KY05 lysate; and High-Phage: RO water with 10^8^ PFU/mL KY05 lysate. ** *n* = 9 per group. *** *n* = 8 per group.

**Table 4:** Effects of KY05 lysate on the jejunum and ileum of laying hens.

Items	Control	Low-Phage	High-Phage	*p* Value
Jejunum				
VH (µm)	659.99 ± 48.60	649.37 ± 39.71	650.54 ± 61.95	0.878
CD (µm)	50.87 ± 6.87	49.74 ± 8.28	50.99 ± 12.70	0.949
VH:CD	13.40 ± 1.99	13.54 ± 2.00	13.44 ± 3.10	0.991
Ileum				
VH (µm)	592.12 ± 35.68	592.90 ± 27.91	593.17 ± 37.68	0.997
CD (µm)	53.76 ± 8.16	51.72 ± 7.12	52.77 ± 3.29	0.784
VH:CD	11.40 ± 1.58	11.73 ± 1.68	11.39 ± 1.16	0.844

Data expressed as mean ± SD (*n* = 10 per group). Statistical comparisons were performed using one-way ANOVA, followed by Tukey’s multiple-comparison test. Differences were considered statistically significant at *p* < 0.05. Control: RO water without KY05 lysate; Low-Phage: RO water with 10^5^ PFU/mL KY05 lysate; and High-Phage: RO water with 10^8^ PFU/mL KY05 lysate.

## Data Availability

The raw data supporting the conclusions of this article will be made available by the authors on request.
